# Oxygen-enhanced MRI MOLLI T1 mapping during chemoradiotherapy in anal squamous cell carcinoma

**DOI:** 10.1016/j.ctro.2020.03.001

**Published:** 2020-03-18

**Authors:** Emma Bluemke, Daniel Bulte, Ambre Bertrand, Ben George, Rosie Cooke, Kwun-Ye Chu, Lisa Durrant, Vicky Goh, Clare Jacobs, Stasya M. Ng, Victoria Y. Strauss, Maria A. Hawkins, Rebecca Muirhead

**Affiliations:** a Institute of Biomedical Engineering, University of Oxford, UK; b Department of Oncology, Oxford University Hospitals NHS Foundation Trust, UK; c Radiotherapy Department, Oxford University Hospitals NHS Foundation Trust, UK; d Department of Oncology, CRUK/MRC Oxford Institute for Radiation Oncology, University of Oxford, UK; e Centre for Statistics in Medicine, NDORMS, University of Oxford, UK; f University College London, UK

**Keywords:** MRI, Oxygen Enhanced MRI (OE-MRI), Chemoradiotherapy, Hypoxia, MOLLI T1-Mapping, Tumour

## Abstract

**Background and purpose:**

Oxygen-enhanced magnetic resonance imaging (MRI) and T1-mapping was used to explore its effectiveness as a prognostic imaging biomarker for chemoradiotherapy outcome in anal squamous cell carcinoma.

**Materials and methods:**

T2-weighted, T1 mapping, and oxygen-enhanced T1 maps were acquired before and after 8–10 fractions of chemoradiotherapy and examined whether the oxygen-enhanced MRI response relates to clinical outcome. Patient response to treatment was assessed 3 months following completion of chemoradiotherapy. A mean T1 was extracted from manually segmented tumour regions of interest and a paired two-tailed *t*-test was used to compare changes across the patient population. Regions of subcutaneous fat and muscle tissue were examined as control ROIs.

**Results:**

There was a significant increase in T1 of the tumour ROIs across patients following the 8–10 fractions of chemoradiotherapy (paired *t*-test, p < 0.001, n = 7). At baseline, prior to receiving chemoradiotherapy, there were no significant changes in T1 across patients from breathing oxygen (n = 9). In the post-chemoRT scans (8–10 fractions), there was a significant decrease in T1 of the tumour ROIs across patients when breathing 100% oxygen (paired *t*-test, p < 0.001, n = 8). Out of the 12 patients from which we successfully acquired a visit 1 T1-map, only 1 patient did not respond to treatment, therefore, we cannot correlate these results with clinical outcome.

**Conclusions:**

These clinical data demonstrate feasibility and potential for T1-mapping and oxygen enhanced T1-mapping to indicate perfusion or treatment response in tumours of this nature. These data show promise for future work with a larger cohort containing more non-responders, which would allow us to relate these measurements to clinical outcome.

## Highlights

• Oxygen-enhanced MRI and T1-mapping explored as prognostic radiotherapy biomarker.
• Significant increase in tumour T1 across patients following chemoradiotherapy.
• Before chemoradiotherapy, OE-MRI showed no significant changes in tumour T1.
• After chemoradiotherapy, OE-MRI showed a significant decrease in tumour T1.
• T1 changes from oxygen-enhanced MRI could indicate change in tumour perfusion.

## 1 Introduction

80% of anal cancers are squamous cell carcinomas (SCC) [1], [2]. In the UK, typical management is either intensity modulated radiotherapy or a two-phase radiotherapy technique, to a total dose of 50.4 Gy, concurrently with Mitomycin and 5-fluorouracil (5FU) chemotherapy [3], [4], [5]. Although outcomes are good with a 5-year disease-free survival of 58–78%, there remains a poor prognostic group with a local relapse rate of over 50% [6], [7]. It is suggested that dose escalation to the primary tumour may result in improved local control [8], however due to complications including faecal incontinence, mucus discharge, vaginal stricturing and rectal bleeding, dose escalation to all patients may result in increased late side effects to patients unnecessarily [8], [9], [10], [11], [12], [13], [14]. Better and early identification of the group of patients in need of radiotherapy treatment intensification would allow clinicians to tailor radiotherapy dose to prevent relapse while mitigating these late side effects.

Ideally, a non-invasive imaging biomarker could predict poor outcome early in the treatment course, to allow an intervention such as dose escalation. Magnetic resonance imaging (MRI) is routinely used in the diagnostic process and could provide a non-invasive method for extracting this biomarker information. In this study, we hypothesize that patients with poor vascularity or hypoxia will correlate with those more likely to be at risk of relapse, and that a technique known as oxygen-enhanced MRI (OE-MRI) can be used to provide indicators of tumour perfusion [15], [16], [17]. In the body, oxygen affects MR contrast by acting as a paramagnetic relaxation agent thus shortening the T1 relaxation time, one of the principal sources of image contrast in MRI [18]. Perfused tumours contain a much higher concentration of oxygen than non-perfused tumours, and that oxygen causes a shortening in T1 relaxation in the tumour tissue [19], [20], [21]. The partial pressure of oxygen within the tumour cannot be determined from the T1 alone however. One method of using this effect to infer perfusion levels is by inducing arterial hyperoxia to further shorten T1 of arterial blood and well-perfused tissues by breathing an increased inspired fraction of oxygen [18], [22], [23]. By acquiring a quantitative map of T1 values in each voxel (known as a “T1 map”) under normal conditions, and then performing the scan a second time while the patient is breathing an increased inspired fraction of oxygen via a face mask, the change in T1 between the air and oxygen T1 maps provides information about both the arterial blood volume in, and perfusion of the tumour [19]. It is by this mechanism that the changes in T1 from this oxygen-enhanced MRI could potentially differentiate perfused tumours from tumours with poor vascularity or hypoxia.

In this study, we quantify the changes in mean T1 between a T1-map acquired at diagnosis and again on fraction 8–10 of chemoradiotherapy and examine whether this oxygen-enhanced MRI response relates to clinical outcome in patients with anal squamous cell carcinoma.

## 2 Methods

### 2.1 Patients

Patients included in this study were 23 trial participants (mean age = 60 years) who underwent radical chemoradiotherapy for anal cancer in Oxford University Hospitals NHS Trust between 2014 and 2017 (ClinicalTrials.gov no.: NCT02145416). All patients had newly diagnosed, histologically confirmed ≥T2N0 anal SCC with no prior treatment and were suitable for radical CRT. Exclusion criteria were contraindications to MR imaging and previous pelvic radiotherapy. This study was approved by National Research Ethics Service Committee South Central Oxford (14/SC/1130) and all patients provided written informed consent.

### 2.2 Treatment

CRT was delivered according to UK-based guidance [25]. A dose of 50.4 Gy or 53.2 Gy was prescribed to the primary tumour depending on TNM stage, 50.4 Gy to involved nodes and a 40 Gy prophylactic dose to non-involved nodes, all in 28 fractions using a simultaneous integrated boost. Three patients were treated within the PLATO study (ISRCTN88455282)[26], two with a dose of 61.6 Gy to the primary tumour while maintaining 40 Gy to the prophylactic nodes, and the other patient with a reduced dose of 41.4 Gy to the primary tumour and 34.5 Gy to elective nodes in 23 fractions with simultaneous integrated boost. Treatment was delivered using intensity modulated radiotherapy (IMRT) or volumetric modulated arc therapy (VMAT). Chemotherapy was Mitomycin 12 mg/m^2^ on day 1 and Capecitabine 825 mg/m^2^ orally twice a day on radiotherapy treatment days. Post CRT tumour response assessment was undertaken three months following completion of CRT using clinical, radiological and, where appropriate, histological analysis. At this point all patients were defined as having had a complete response or having persistent disease.

#### 2.2.1 MRI protocol

Patients were scanned on a GE Discovery MR750 3T MR scanner with a GE 8-channel Torso Array (GE Healthcare Milwaukee, WI, USA) using a flat-topped couch to reproduce the radiotherapy treatment position. Following the acquisition of a T2-weighted image (FRFSE sequence, TR 3700s, TE 133.50s, slice thickness 3.0 mm, slice gap 0.3 mm, matrix size 512 × 512, field of view 240x240, 21 slices), the Modified Look-Locker Inversion Recovery (MOLLI) [24] T1-mapping sequences (FIESTA sequence, slice thickness 5.0 mm, resolution 1.7 mm × 1.7 mm, FOV = 380 mm, 11 inversion times, flip angle = 35°, TR = 3000 ms, TE = 120 ms) were acquired in a single transverse slice through the tumour volume. The MOLLI scans were acquired while the patient was breathing air and then again when given 100% oxygen to breathe via an Intersurgical EcoLite high concentration oxygen mask (Intersurgical Ltd, Berkshire, UK). Scanning was performed only once the end tidal oxygen levels reached 70%, measured using a Respiratory Gas Analyzer ML206 and and a PowerLab 4/26 (ADInstruments Ltd, Oxford, UK). This imaging procedure was performed prior to treatment (‘visit 1’), and after fraction 8–10 of radical chemoradiotherapy (‘visit 2’).

#### 2.2.2 Region of interest analysis

**Fig. 1:**
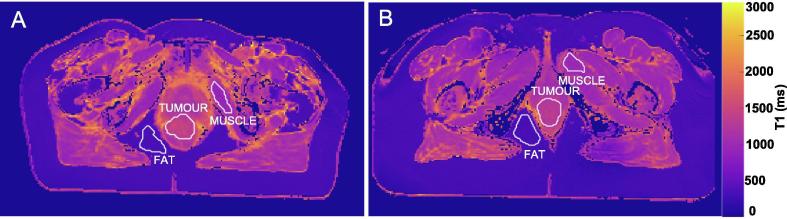
Examples of the tumour, fat, and muscle regions of interest used in this study, shown on **(A)** patient H and **(B)** patient A.

A tumour region of interest (ROI) was delineated by an experienced oncologist for all primary tumours and any lymph nodes >2 cm on high-resolution T2-weighted images and nonlinearly registered to the T1-map. Due to the resolution and slice thickness differences between the MOLLI T1 maps and T2-weighted images, as well as mild spatial distortions in the MOLLI due to the high speed acquisition, there is not a direct 1:1 mapping between the T2-weighted images and T1 maps and thus registration was needed. Registration was implemented using MATLAB’s intensity-based registration algorithm (MATLAB R2016, MathWorks, Natick, MA). Patient response to treatment was assessed 3 months following completion of chemoradiotherapy. For use as control ROIs, regions of fatty tissue and muscle tissue were also delineated (examples shown in Fig. 1). The muscle and fatty tissue regions were selected to be approximately the same number of voxels as the tumour ROI and as close to the tumour ROI as possible, thus expected to have received some dose of radiation.

For each region of interest, the histogram distribution of T1 values was examined, checking whether there was a normal distribution or, for example, a bimodal distribution possibly representing two populations of tissue in the ROI. In all cases, the distribution was normal, thus indicating that a comparison of means was suitable to describe changes in the ROIs. The changes in mean T1 was assessed with a paired two-tailed *t*-test (a = 0.05) considering 3 cases: the change in T1 (1) from visit 1 to visit 2, (2) breathing air versus oxygen on visit 1, and (3) breathing air versus oxygen on visit 2. The tumour ROIs contained an average of 265 ± 187 voxels, and patients with tumour ROIs containing <50 voxels were excluded from the analysis. Out of the 54 total MOLLI images acquired, 5 were excluded from analysis due to containing <50 tumour voxels within the MOLLI slice. The largest tumour ROI was 728 voxels. If the T1 map was of insufficient quality due to excessive patient motion or scanner artefacts, the patient was excluded from the analysis.

In addition, we investigated the possibility that the native T1 could correlate to the amount of change seen from breathing oxygen in either visit. Pearson correlation was used to estimate the correlation between: the tumour T1 while breathing air in visit 2 and the decrease in T1 from oxygen in visit 2 (calculated as T1_air_ − T1_oxygen_), the tumour T1 while breathing air in visit 2 and % change in T1 in visit 2 (calculated as (T1_air_ − T1_oxygen_)/T1_air_), and the tumour T1 while breathing air in visit 1 and either of these measures in visit 2.

At the outset of the study, two more analyses were intended: the patient outcomes, complete response or persistent disease, were to be correlated to (4) the T1 on visit 1 on air and (5) the change in T1 from oxygen on visit 1. However, there was only one patient whose data passed the quality metrics who did not respond to therapy, so it was not possible to perform these final two analyses.

## 3 Results

Out of the 23 patients included in the study, a T1 map was successfully acquired on visit 1 for 11 patients. By visit 2, following chemoradiotherapy, some tumours were too small to include in the analysis, resulting in a visit 2 T1 map for 7 of those 12 patients. In total, oxygen-enhanced T1 maps were successfully acquired for 9 patients on visit 1 and 8 patients on visit 2. Table 1 contains a summary of the resultant viable ROIs used in this analysis. Out of the 12 patients from which we successfully acquired a visit 1 T1-map, only 1 patient did not respond to treatment and no T1 maps from visit 2 were available for that patient. A table of characteristics of all patients included and excluded in the analysis is included in the Supplementary Materials.

**Table 1:** A summary of the patients included in this analysis. The marking of “X” indicates a T1-map was successfully acquired and the tumour ROI was large enough to include in the analysis.

Patient		A	B	C	D	E	F	G	H	I	J	K	L	M
Visit 1	Air	X	X	X	X	X	X		X	X	X	X	X	X
	O_2_	X	X	X	X	X	X		X	X	X			
Visit 2	Air	X	X	X		X	X	X	X	X				
	O_2_	X	X	X		X	X	X	X	X				

**Fig. 2:**
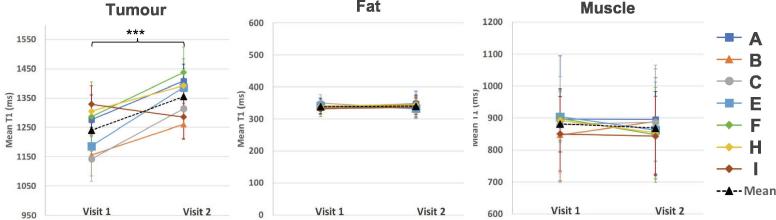
The mean T1 in the tumour, fat and muscle ROIs across the two visits. The patients received 8–10 fractions of chemoradiotherapy between the two visits. There was a significant increase in T1 of the tumour ROIs across patients (paired *t*-test, p < 0.001). There were no significant changes in T1 in the control ROIs of fat and muscle tissue. The error bars indicate the standard deviation of the pixel values in the ROI.

The change in the mean T1 in the tumour, fat and muscle ROIs were compared across the two visits (Fig. 2). There was a significant increase in T1 of the tumour ROIs across patients following the 8–10 fractions of chemoradiotherapy (paired *t*-test, p < 0.001, n = 7), while there were no significant changes in T1 in the control ROIs of fat and muscle tissue. The largest change in mean T1 in the tumour ROI seen was an increase of 200 ms, and the mean change across patients was an increase of 115 ms.

**Fig. 3:**
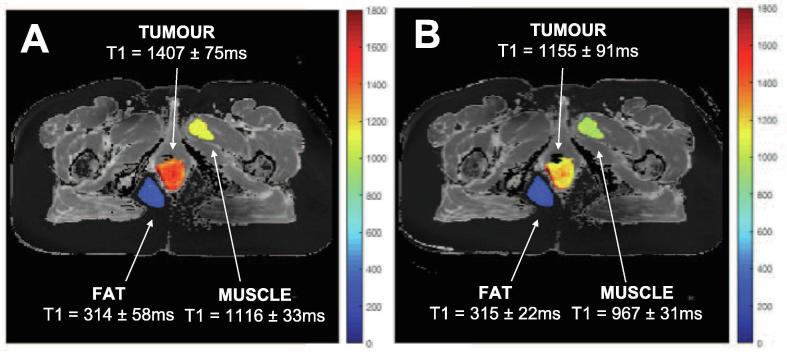
Example of the changes in T1 in the tumour, fat, and muscle regions of interest from patient A on visit 2. The ROI sections of the T1-map when breathing **(A)** air and **(B)** 100% oxygen are shown overlayed with a jet colourmap on the respective grayscale T1-map for a visual aid. Each ROI is labelled with the mean T1 ± standard deviation.

**Fig. 4:**
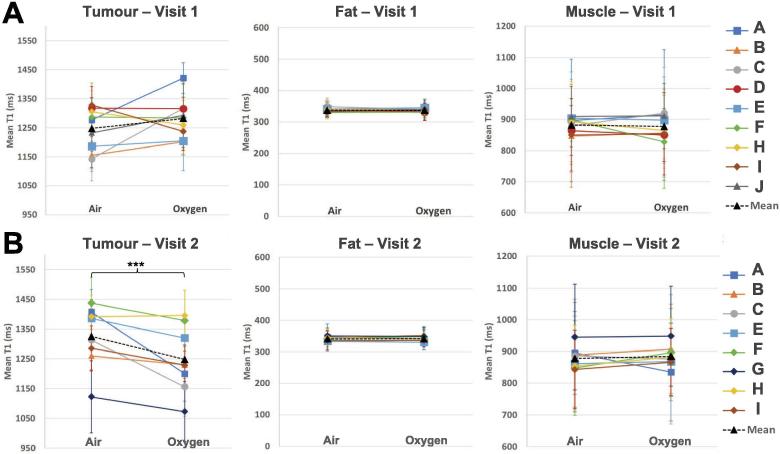
The mean T1 in the tumour, fat and muscle ROIs in the T1-map taken while the patient was breathing air versus 100% oxygen. **(A)** There were no significant changes in T1 across patients from breathing oxygen in visit 1. **(B)** There was a significant decrease in T1 of the tumour ROIs across patients when breathing 100% oxygen (paired *t*-test, p < 0.001). In all cases, there were no significant changes in T1 in the control ROIs of fat and muscle tissue. The error bars indicate the standard deviation of the pixel values in the ROI.

The mean T1 in all ROIs taken while the patient was breathing air versus 100% oxygen were compared, in each visit separately (patient example shown in Fig. 3, all patients summarized in Fig. 4). In visit 1, prior to receiving chemoradiotherapy, there were no significant changes in T1 across patients from breathing oxygen (paired *t*-test, p < 0.11, n = 9). In visit 2, after receiving 10–12 fractions of chemoradiotherapy, there was a significant decrease in T1 of the tumour ROIs across patients when breathing 100% oxygen (paired *t*-test, p < 0.001, n = 8). In all cases, there were no significant changes in T1 in the control ROIs of fat and muscle tissue. In visit 2, the largest change in mean T1 in the tumour ROI from breathing oxygen was a decrease of 207 ms, and the mean change across patients was a decrease of 78 ms.

A Pearson correlation showed no significant correlation between: the tumour T1 while breathing air in visit 2 and the decrease in T1 from oxygen in visit 2 (calculated as T1_air_ − T1_oxygen_), the tumour T1 while breathing air in visit 2 and % change in T1 in visit 2 (calculated as (T1_air_ − T1_oxygen_)/T1_air_), or the tumour T1 while breathing air in visit 1 and either of these measures in visit 2.

**Fig. 5:**
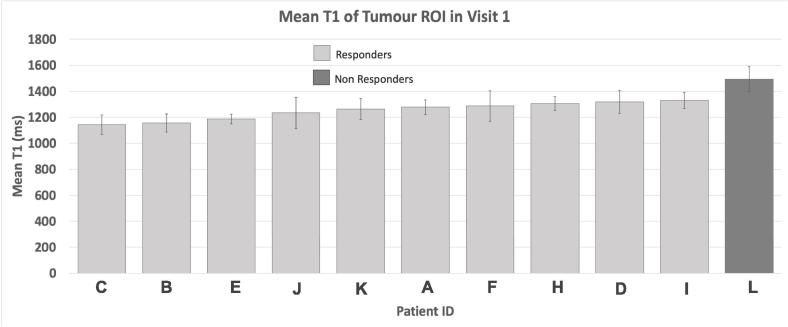
The mean T1 of the tumour ROI in all patients on the first visit, prior to receiving any chemoradiotherapy. Patients who responded to the chemoradiotherapy over the trial are shown in light grey, patients who did not respond to treatment are shown in dark grey. The error bars indicate the standard deviation of the pixel values in the ROI.

The mean tumour T1 measurements at diagnosis are shown in Fig. 5. It can be noted that the 1 non-responding tumour had a higher mean T1 (1493 ms) than all of the responding tumours (group mean = 1249 ± 67 ms, maximum = 1328 ms, minimum = 1142 ms).

## 4 Discussion

In this study, we quantify the changes in mean T1 between a T1-map acquired at diagnosis and again following chemoradiotherapy and examine whether the native (while breathing air) T1 or oxygen-enhanced MRI T1 maps relate to clinical outcome in patients with anal squamous cell carcinoma. Out of the 12 patients from which we successfully acquired a visit 1 T1-map, only 1 patient did not respond to treatment. We demonstrate feasibility and potential for T1-mapping and OE-MRI to indicate perfusion in future work with larger cohort containing more non-responders, which would allow us to relate these measurements to clinical outcome. As the numbers are small a clinical correlation cannot be made.

Following chemoradiotherapy, there was a statistically significant increase in tumour T1 across the 7 patients (Fig. 2), all of which showed a complete clinical response at 3 months. This change in T1 is not unexpected, since the tumours were treated aggressively between visits which would cause changes in the cellular and biochemical environment. It is important to note that all 7 patients responded to treatment, so the changes in T1 observed at visit 2 (midway through chemoradiotherapy) are likely due to changes in the cellular and tumour microenvironment that were occurring and ultimately resulting in complete response in the patient.

Since the tumours were often smaller at visit 2 due to treatment response, it is possible that the tumour remaining in visit 2 is just the more aggressive section, and perhaps always had a different T1, which could explain this change. However, we hypothesize that this is not the case, since no bimodal distributions or multiple populations were seen in histograms when analyzing all of the ROIs, suggesting this change in T1 does represent a true change in the tumour biochemistry and/or physiology.

In addition, the changes seen from oxygen-enhancement changed greatly following chemoradiotherapy. Before chemoradiotherapy, OE-MRI showed no significant changes in tumour T1, however after receiving chemoradiotherapy, OE-MRI showed a significant decrease in tumour T1 (p < 0.001) (Fig. 4). The largest change in mean T1 in the tumour ROI from breathing oxygen was a decrease of 207 ms, and the mean change across patients was a decrease of 78 ms. The magnitude of T1 shortening seen across the patients in this study was very similar to the changes observed by O’Connor et al. in a set of 10 patients with a variety of tumour types, where the greatest change was a decrease of 136 ms and mean change was a decrease of 59 ms [15]. This change in oxygen response following treatment could be indicative of a change in perfusion in the tumour. It is again important to note that all 8 patients in this comparison showed a clinical response at 3 months, so all tumours were undergoing changes that ultimately resulted in patient recovery. A further change in tumour T1 from OE-MRI, however, also indicates that the oxygen was able to reach the tumour, thus indicating that there is perfusion in the tumour. Inversely, however, one cannot confidently conclude that no change from OE-MRI confirms a lack of perfusion; oxygen is a biologically active molecule which will be consumed by the body and even change the physiology by causing vasoconstriction. It is hypothetically possible that in visit 1, additional oxygen did reach the tumour but was consumed rapidly by the possibly hypoxic, oxygen-starved tissue.

As shown in Fig. 5, the 1 non-responding tumour had a higher mean T1 than all of the responding tumours. It is noteworthy that the non responder having a higher T1 than the responders, while not ruling out the initial T1 as a biomarker, provides no proof that it is a biomarker. We note also that an increase in T1 has occurred in all non-responders from the treatment itself. However, it is important to consider that the conditions determining the T1 in the tumour prior to treatment and the treatment induced change in T1 can result from dramatically different origins. In addition, no conclusions can be drawn from only one patient sample.

While this study offers promising results, the technique has some limitations which warrant discussion. First, due to loss of data from a combination of patient motion, trial withdrawal, and tumour shrinkage, this study had a relatively small sample size, and no results could be correlated to clinical outcome. Secondly, the T1-mapping method used, MOLLI, currently provides only single-slice data, limiting the analysis to one region of the tumour. As tumours can be highly heterogeneous, it is possible that this analysis misses large changes that were occurring in other regions, and in addition, although care was taken to select the same region of the tumour on the second visit, it is possible that the slices are analyzing slightly different slices of the tumour on each visit. This limitation could be mitigated by the use of a 3D T1 mapping method such as the variable flip angle method [27], [28], although this much slower technique can also suffer from patient movement requiring more complex image registration during analysis.

A final limitation that warrants discussion is that the T1 contrast provided by increased fractions of inspired oxygen tends to decrease as magnetic field strength increases [29], [30]. In this study, performed at 3 T, the largest change in mean tumour T1 from OE-MRI was a decrease of 207 ms, with a mean decrease across patients of 78 ms. Although this resulted in a detectable and significant change, future studies may benefit from using lower field strengths such as 1.5 T.

Lastly, we investigated the possibility that the native T1 could correlate to the amount of change seen from breathing oxygen, however there was no significant correlation between these values on either visit. This is not unexpected, since tumours are highly varied and heterogeneous between patients and the mean T1 will be affected by a variety of factors, not only the perfusion of the tumour.

In conclusion, this clinical trial data demonstrates the feasibility and potential for T1-mapping and oxygen enhanced T1-mapping to indicate perfusion or treatment response in tumours of this nature. The data show promise for future work with a larger cohort which would likely result in more non-responders, allowing us to relate these measurements to clinical outcome.

## Declaration of Competing Interest

The authors declare that they have no known competing financial interests or personal relationships that could have appeared to influence the work reported in this paper.
