# Supplementary material for: Oxygen-enhanced MRI MOLLI T1 mapping during chemoradiotherapy in anal squamous cell carcinoma
Source: Clin Transl Radiat Oncol. 2020 Mar 18;22:44–9. doi: 10.1016/j.ctro.2020.03.001 (PMC7082428; doi:10.1016/j.ctro.2020.03.001)
Supplement: Supplementary data 1 [file mmc1.pdf]

Baseline factor	Trial Patients included in analysis	Trial patients excluded in analysis
**Sex (%)**		
Female	10 (83%)	13 (76%)
Male	2 (17%)	4 (24%)
**Age at enrolment (years) Median (LQ, UQ)**		
Age		
**Histologic grade (%)**		
Well differentiated	0 (0%)	1 (5%)
Moderately differentiated	5 (42%)	10 (59%)
Poorly differentiated	1 (8%)	3 (18%)
Undifferentiated/ Level of differentiation not assessed.	6 (50%)	3 (18%)
**Lymphovascular invasion (%)**		
Absent	6 (50%)	4 (24%)
Present	1 (8%)	5 (29%)
Unknown	5 (42%)	8 (47%)
**ECOG performance status (%)**		
0	8 (67%)	14 (82%)
1	4 (33%)	0 (0)
Unknown	0 (0)	3 (18%)
**Tumour site (%)**		
Anal canal	9 (75%)	16 (94%)
Anal verge	2 (17%)	1 (6%)
Other	1 (8%)	0 (0%)
**T stage (%)**		
T2	8 (67%)	10 (59%)
T3	1 (8%)	4 (23%)
T4	3 (25%)	1 (6%)
Unknown	0 (0)	2 (12%)
**N stage (%)**		
N0	0 (0%)	7 (41%)
N1	6 (50%)	2 (12%)
N2	6 (50%)	1 (6%)
N3	0 (0%)	5 (29%)
Unknown	0 (0%)	2 (12%)
**M stage (%)**		
M0	0 (0%)	0 (0%)
M1	12 (100%)	15 (88%)
Unknown	0 (0%)	2 (12%)

Supplementary Table 1*.* Baseline characteristics of recruited patients (N=23).

Patient ID	Visit 1		Visit 2	
	Air	O_2	Air	O_2
**101-109**	MOLLI not acquired as imaging was not included in study protocol yet			
**110**	Patient withdrew from trial			
**111**	Not acquired	O2 not in protocol	✓	O2 not in protocol
**112 (L)**	✓	O2 not in protocol	Error in MOLLI acquisition	O2 not in protocol
**113 (K)**	✓	O2 not in protocol	Scanner crashed	O2 not in protocol
**114**	Tumour obstructed by bowel gas		Tumour obstructed by bowel gas	
**115**	MOLLI slice missed tumour		Tumour obstructed by bowel gas	
**116**	Tumour ROI too small		Tumour ROI too small	
**117 (J)**	✓	✓	Tumour ROI too small	
**118**	Patient withdrew from trial			
**119**	Patient withdrew from trial			
**120 (I)**	✓	✓	✓	✓
**121**	Patient withdrew from trial			
**122 (H)**	✓	✓	✓	✓
**123 (G)**	Tumour obstructed by bowel gas		✓	✓
**124 (F)**	✓	✓	✓	✓
**125 (E)**	✓	✓	✓	✓
**126 (D)**	✓	✓	MOLLI not acquired - scanner crashed	
**127 (C)**	✓	✓	✓	✓
**128 (B)**	✓	✓	✓	✓
**129 (A)**	✓	✓	✓	✓

Supplementary Table 2. A table of characteristics of all patients included and excluded in the analysis. Reasons include patient withdrawal (4 patients), the tumour ROI being obstructed due to bowel gas passing at the time of imaging (causing 8 images in total to be excluded), and the MOLLI slice encompassing too small of a region of the tumour (this was an issue in 4 patient sessions). In addition, the scanner crashed twice over the duration of the study, leading to missing data for 2 subjects in their second visit.
